# Pilot Study of Acupuncture’s Antispasmodic Effect on Upper Gastrointestinal Tract during Endoscopic Submucosal Dissection for Early Gastric Cancer: Controlled Clinical Trial

**DOI:** 10.3390/jcm10143050

**Published:** 2021-07-09

**Authors:** Masao Suzuki, Naoto Ishizaki, Takumi Kayo, Taiga Furuta, Ryo Igarashi, Takumi Maki, Koki Hoshi, Akane Yamabe, Mariko Fujisawa, Akira Funakubo, Tadamichi Mitsuma, Atsushi Irisawa, Goro Shibukawa

**Affiliations:** 1 Department of Kampo Medicine, Aizu Medical Center, Fukushima Medical University School of Medicine, Aizuwakamatsu, Fukushima 969-3492, Japan; k-takumi@fmu.ac.jp (T.K.); tigerinatora@gmail.com (T.F.); tmitsuma@fmu.ac.jp (T.M.); 2 Course of Acupuncture and Moxibustion, Faculty of Health Sciences, Tsukuba University of Technology, Kasuga, Tsukuba 305-0821, Japan; ishizaki@k.tsukuba-tech.ac.jp; 3 Department of Gastroenterology, Aizu Medical Center, Fukushima Medical University School of Medicine, Aizuwakamatsu, Fukushima 969-3492, Japan; ryo.igarashi@keio.jp (R.I.); m06094tm@jichi.ac.jp (T.M.); k.hoshi.529@gmail.com (K.H.); yamaaka0110@yahoo.co.jp (A.Y.); george-monkey@kxa.biglobe.ne.jp (M.F.); akira726@fmu.ac.jp (A.F.); irisawa@dokkyomed.ac.jp (A.I.); goro4649@aol.com (G.S.); 4 Department of Organoid Medicine, Keio University School of Medicine, Shinjuku, Tokyo 160-8582, Japan; 5 Internal Medicine, Fukushima Prefectural Minamiaizu Hospital, Minamiaizu-gun, Fukushima 967-0006, Japan; 6 Department of Gastroenterology, Dokkyo Medical University School of Medicine, Shimotsuga, Tochigi 321-0293, Japan; 7 Division of Gastroenterology and Hepatology, Department of Medicine, Nihon University School of Medicine, Itabashi, Tokyo 173-8610, Japan

**Keywords:** acupuncture, antispasmodic, upper gastrointestinal tract, endoscopic submucosal dissection

## Abstract

A prospective study was conducted in patients with early-stage gastric cancer to determine the efficacy and safety of acupuncture stimulation as an antispasmodic compared with conventional medication during the procedure of endoscopic submucosal dissection (ESD) of the upper gastrointestinal tract. This study was a prospective single blinded quasi-randomized controlled trial. Seventy-three patients who were scheduled to undergo ESD for gastric cancer at Aizu Medical Center between 19 February 2016 and 30 June 2016 were assessed for eligibility for the study. Sixty out of 73 patients were included in the study and assigned into two intervention groups: medication group (MG) and acupuncture group (AG). Ease of the procedure was evaluated using modified NIWA classification (MNC) by endoscopist considering the frequency and amplitude of the upper gastrointestinal peristalsis. For the statistical analysis, Mann–Whitney test was used to compare the differences of MNC values (baseline and end of procedure) between two groups. The difference of MNC found in the AG (−2.00 (−3.0 to −2.0)) was significantly greater than that in the MG (−1.00 (−2.0 to −1.0), *p* < 0.0001, Mann–Whitney test). We consider that acupuncture to the abdomen could be an alternative antispasmodic method during upper gastrointestinal endoscopic procedure.

## 1. Introduction

Endoscopic submucosal dissection (ESD) is a procedure that enables en bloc resection of gastric neoplastic lesions, and ESD is the first-line treatment for early-stage gastrointestinal cancer [1,2].

Adverse events during ESD procedure such as perforation and bleeding may be caused by poor visualization of the resection area besides fibrosis or scars [3,4,5]. As the gastric peristalsis especially hinders the field of vision and makes the procedure difficult, it is important to inhibit gastric peristalsis from the early phase of the ESD procedure.

Normally, antispasmodic agents such as “anticholinergics” and “glucagon” are used, but for patients with underlying diseases such drugs may be contraindicated or require careful administration. According to a nationwide survey on the adverse events during gastroendoscopy in Japan, cases of accidental events due to the use of antispasmodic agents were observed [6]. Furthermore, as the number of patients with lifestyle-related diseases increases, we may see more situations in which such antispasmodic drugs are not preferable in gastrointestinal procedure. There is an increased desire to develop an alternative method to antispasmodic agents.

Acupuncture is a non-pharmacological treatment with less adverse events than that observed in conventional medicine. Of various effects of acupuncture on the upper and lower gastrointestinal organ reported [7], acupuncture stimulation at Zhongwan (CV12) causes relaxation in stomach through the autonomic nervous system [8,9]. Although such antispasmodic effect of acupuncture stimulation has been considered fairly safe, its usefulness for ESD procedure has not been clearly demonstrated.

Our purpose is to verify whether acupuncture stimulation to CV12 can be an alternative treatment for antispasmodic agents in ESD procedure.

## 2. Materials and Methods 

### 2.1. Study Design

We prospectively compared acupuncture with conventional antispasmodic agent in terms of the antispasmodic effect on the stomach and the safeness during ESD procedure for early-stage gastric cancer patients. The study design is quasi-randomized parallel group study. It was shown in the detailed protocol of this study (Supplement Materials; The study protocol (File S1)).

### 2.2. Patients

Patients who were scheduled to undergo ESD for gastric cancer at Aizu Medical Center between 24 February 2016 and 30 June 2016 were screened for eligibility for the study. 

Inclusion criteria were:

1. Those who were age >40 years and diagnosed as early-stage gastric cancer, irrespective of *H. pylori* infection. Early-stage gastric cancer was pathologically diagnosed using endoscopy prior to ESD [2]. The criteria of early-stage gastric cancer were: (1) An intramucosal intestinal-type cancer without ulcerative lesion, regardless of tumor size; (2) intramucosal intestinal-type cancer with ulcerative lesion, ≤3 cm in size; and (3) intramucosal diffuse type cancer ≤2 cm in size without ulcerative lesion.
2. Those who were in stable condition without any symptoms of infectious diseases such as fever or respiratory symptoms, showed no rapid deterioration of cancer related symptoms and with no changes in the medication at least a week prior to the date of the ESD.
3. Patients who had sufficient cognitive ability to understand the study protocol.

Exclusion criteria were patients with (1) history of partial gastrectomy; (2) severe uncontrollable diabetes or severe cardiovascular disease; (3) advanced stage gastric cancer; and (4) lack of informed consent. 

Written informed consent was obtained from all the participating patients.

This study was performed in accordance with the Declaration of Helsinki and its amendments, and the Guidelines for Good Clinical Practice for Epidemiological Studies and Clinical Research issued by the Japanese Ministry of Health. This study also followed the Standards for Reporting Interventions in Controlled Trials of Acupuncture (STRICTA) guidelines [10], and the study protocol was approved in advance by the Institutional Review Board of the Fukushima Medical University of Medicine Science (Number: 2581, Approval date 17 December 2015). This trial was registered in the University Hospital Medical Information Network, Clinical Trials Registry (UMIN000021065).

### 2.3. Patients Registration Assignment of the Subjects

The first 10 patients were assigned to the acupuncture group (AG), the next 10 patients were assigned to the medication group (MG), then the next 10 patients to the AG, and so on. This alternate assignment procedure was repeated until 60 cases (30 cases in each group) were enrolled.

### 2.4. ESD Procedure

Patients were administered with high concentration oxygen (2 L/h), and then received a slow initial intravenous bolus of 0.15 to 0.30 mg/kg of Midazolam. In cases of insufficient sedation, additional intravenous boluses of 0.5 mg/kg of propofol were slowly administered until the patients were sedated, as determined by the Ramsay sedation score of 5–6. Most patients received 7.5 mg of pentazocine as an analgesic agent at the start of the ESD and at 60 min intervals thereafter during the procedure.

When a patient seemed to be in discomfort or exhibited restlessness following verbal stimulation, an additional 10 mg of propofol or/and 7.5 mg of pentazocine were given as a bolus injection. 

ESD was carried out by any one of six gastroenterologists with more than 5 years of experience with a single channel endoscope (GIF-H290Z; Olympus, Tokyo, Japan). We used a dual knife (KD-650; Olympus, Tokyo, Japan) or/and IT knife2 (KD-611L; Olympus, Tokyo, Japan) as the cutting device, and an electrical current was applied using an electrosurgical generator (VIO300D; ERBE Elektromedizin GmbH, Tubingen, Germany). Visible vessels were heat-coagulated using hemostatic forceps (Coagrasper G; FD-412LR, Olympus, Tokyo, Japan).

All the necessary procedures for ESD and sedation were performed by the endoscopists according to the guidelines [2,11].

### 2.5. Interventiona and Blinding

Initial evaluation of the peristaltic movement was observed at the antrum including the pyloric ring. In case gastrointestinal tract peristalsis was found, medication or acupuncture stimulation was given when the Niwa classification was not less than 2 [12].

For MG, glucagon 0.5 mL (1 mg/mL) was administered as anticonvulsant after confirmation of the peristaltic movement. Additional glucagon 0.5 mL (1 mg/mL) was administered when the peristalsis persisted after the initial administration. 

**Figure 1:**
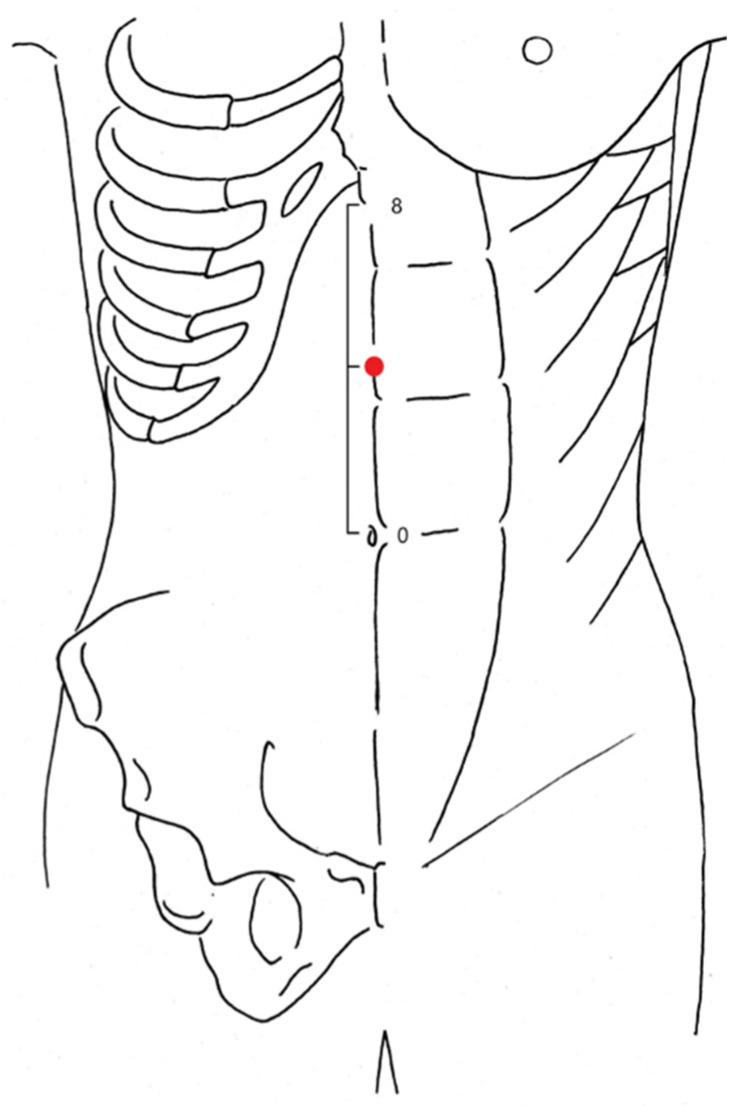
Zhongwan (CV12) (indicated as a red dot) is located on the anterior midline, 4 cun above the umbilicus. 1 cun is equivalent to 30.3 mm.

For AG, acupuncture was performed by any one of two licensed acupuncturists with more than 5 years of clinical experience and were aware of assignments of the intervention. Zhongwan (CV12) is located on the midline, between the lower end of the xiphoid process and the navel. (Figure 1) [13]. After confirmation of peristalsis of the stomach, an acupuncture needle was inserted into the Zhongwan (CV12) acupoint to penetrate the rectus abdominis muscle (Linea alba), and then turned clockwise to wrap the muscle around the needle. After that, additional manipulation of thrusting and withdrawing of the needle was alternately repeated for 3 min. The manipulation was stopped when the peristaltic movement of the stomach was subsided and resumed whenever the movement was observed. The procedure was carried out through the entire ESD procedure. For the acupuncture stimulation, disposable sterilized stainless-steel acupuncture needles (0.25 mm diameter, 40–60 mm long, Seirin, Inc., Shizuoka, Japan) were used. 

The patients were not informed in advance which group they were going to be assigned in but were informed about it after ESD. In addition, the endoscopist and the operator knew in advance about which procedure (acupuncture or medication) was to be taken. On the other hand, the biostatisticians performed the analysis with the patient assignment information masked.

### 2.6. Measures

#### 2.6.1. Primary Outcome Measure

The ease of procedure according to the state of the upper gastrointestinal peristalsis during ESD was evaluated by the endoscopist using the Modified NIWA classification (MNC) (Table 1) [12,14].

#### 2.6.2. Secondary Outcome Measures

Visual analogue scale (VAS) was also utilized in order to evaluate the ease of the procedure. VAS is a 100 mm horizontal scale of which the left end (0 mm) represents “the easiest” and the right end (100 mm) “the most difficult”. 

MNC and VAS were evaluated twice for each procedure. The first was after inserting an endoscope and marking the tumor (baseline), and the second was when the procedure was completed (endpoint). The primary outcome measures and secondary outcome measures were obtained by independent evaluators.

#### 2.6.3. Other Outcome Measures

Other outcome measures related to complications were:

1. Bleeding (intraoperative and postoperative), perforation (intraoperative and delay) due to ESD. Here, bleeding during the procedure is defined as when bleeding continued even after coagulation with normal hemostats, and when clipping or alcohol local injection had to be performed. Bleeding after treatment is defined as when hemostasis at the excised site had to be performed by upper gastrointestinal endoscopy because hematemesis, melena, or shock was observed.
  Perforation was defined as when a perforation was clearly confirmed during the operation and endoscopic clip closure was performed. Delayed perforation was defined as when peritonitis was suspected, and free air was found by abdominal X-ray or CT scan.
2. Additionally, blood pressure fluctuation was monitored during procedure. Blood pressure fluctuations were defined as greater than 30% increase or decrease from the baseline value (base line value is the blood pressure during a 10 min rest before the procedure) and such increase or decrease last for 3 min or more within 5 min after intervention with antispasmodics or acupuncture.
3. Presence or absence of aspiration pneumonia after treatment was evaluated comprehensively by not only respiratory symptoms (cough, sputum, dyspnea, chest pain), and fever, but also findings by images of chest X-ray or chest CT, and sputum examination and blood culture.
4. In accordance with the ASA Physical Status Classification System (ASA-PS) [15], the operation time, anesthesia time, total amount of sedative medication, and resection area were also evaluated. The resection area was calculated by ImageJ software, version 1.6.0 (NIH, Bethesda, MD, USA).

### 2.7. Sample Size and Statistical Analysis

Statistical analysis of this study was conducted by independent two biostatisticians (K.T., I.N.). In order to avoid bias, the biostatisticians performed the statistical analysis with grouping information masked.

The sample size of this study was computed based on the expected value of the primary outcome, which is the change in the MNC at the end of the ESD. Previous studies have reported that gastric peristalsis was suppressed by about 1.8 points in MNC when acupuncture was applied to the abdomen (CV12) during upper gastrointestinal endoscopy procedure. Furthermore, the difference of MNC change between the acupuncture group and the medication group was 0.2 points [16]. As the previous study was a non-RCT and it found no significant difference between medication group and acupuncture group, we took a conservative estimation of MNC difference as 1 and the standard deviation as 1. 

Thus, the required sample size was calculated to be 46 patients (MG 23 and AG 23) to detect a minimal difference of MNC means = 1 with SD = 1 at a significance level of 0.05 (α = 0.05) with a power of 0.9 (β = 0.1). In this study, 30 patients per group were recruited in anticipation of a certain dropout.

This study was conducted with the full analysis set (FAS), therefore participants who were not able to complete the study were excluded from the analysis. All the data were made into a dataset by an independent data entry operator.

The measured continuous and ordinal variables were presented as Medians and interquartile ranges (IQR), and count variables as numbers and percentages. Clinical baseline characteristics data were compared between the two groups by the Mann–Whitney test for continuous variables, and the chi-square test for count variables whenever appropriate. The main analysis, the difference between baseline and endpoint was compared using Mann–Whitney test for ordinal variables and continuous variables, and the chi-square test for count variables whenever appropriate. All statistical analyses were performed using STATA software, version 15 (Stata Corp., College Station, TX, USA).

## 3. Results

### 3.1. Study Population 

**Figure 2:**
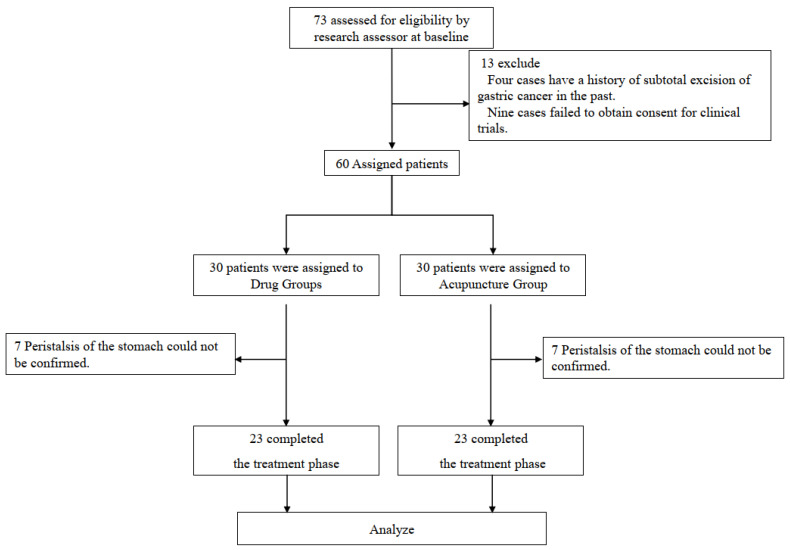
Flow chart of the study participants.

During the period from 24 February 2016 to 30 June 2016, 73 patients with the clinically diagnosed early-stage gastric cancer were found to meet the inclusion criteria. Among them, 13 people were excluded: four cases had a history of subtotal excision of gastric cancer in the past and 9 cases did not consent to the clinical trial. Sixty patients out of these 73 agreed to participate in the study. Of those participants, 14 were unable to complete the study because there was no peristalsis in the stomach (7 in the MG and 7 in the AG) (Figure 2). 

The baseline characteristics of the patients in each group are shown (Table 2).

### 3.2. Primary Outcome (MNC) 

**Figure 3:**
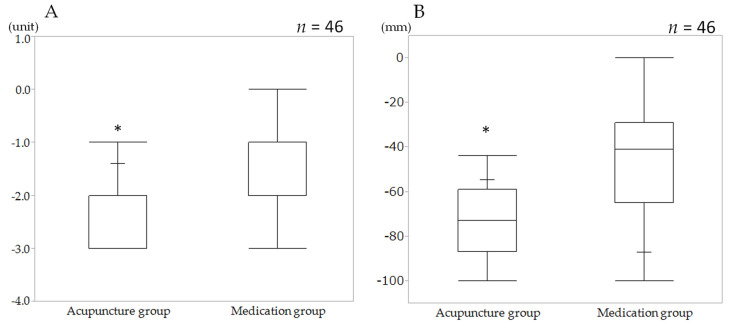
The difference in the antispasmodic action of the upper gastrointestinal tract before and after medication and acupuncture. (**A**) Modified NIWA classification. (**B**) Visual analogue scale. The bottoms and tops of the white boxes represent the 25th and 75th percentiles, respectively, and the lines in the middles represent the 50th percentiles (medians). The whiskers (lines that extend out of the tops and bottoms of the black boxes) represent the highest and lowest values within 1.5 times the interquartile range. * *p* < 0.0001, Mann–Whitney test. Visual analogue scale (VAS).

The MNC decreased from 4.00 (3.0 to 4.0) unit at the baseline to 1.00 (1.0 to 1.0) unit after ESD in the AG, and 4.00 (3.0 to 4.0) unit to 2.00 (2.0 to 3.0) unit in the MG, respectively. The difference in the MNC in the AG (−2.00 (−3.0 to −2.0)) unit was significantly greater than that in the MG (−1.00 (−2.0 to −1.0) unit; by Mann–Whitney test, *p* < 0.0001) (Table 3, Figure 3A).

### 3.3. Secondary Outcome (VAS) 

The difference of VAS in the AG −73.00 (−87.0 to −59.0) mm was significantly greater than that in the MG −41.00 (−65.0 to −29.0) mm (Mann–Whitney test, *p* < 0.0001). (Table 3, Figure 3B) The main data for this study was shown in Supplement Materials (data set of primary and secondary outcome measures (File S2)).

### 3.4. Procedure Time and Area of Resected Specimen

The procedure time and anesthesia time were significantly longer in the AG than in the MG, but the resected area was also greater in the AG. On the one hand, the amount of anesthetic used during procedure was not significantly different between the two groups (Table 4).

### 3.5. Procedure Complications

The numbers of complications such as bleeding, perforation, emergency surgery, and aspiration pneumonia were few in both groups and were not significantly different. 

However, with regard to blood pressure fluctuations, the number of patients who had the incidence was significantly less in the AG (2 (8.70%)) than in the MG (14 (60.86%)), *p* = 0.001 (Table 4). 

The median (IRQ) amount of glucagon used in the MG was 0.5 (0.5 to 1.0) ml. Additionally, the number of patients who received additional administration of glucagon due to recurrence of peristalsis during the procedure was seven. No serious adverse events due to acupuncture stimulation were reported.

## 4. Discussion

This is the first study that evaluated the usefulness of acupuncture for suppressing gastric peristalsis during ESD procedure. For patients, successful gastric cancer surgery is of paramount importance. For that purpose, it is important to minimize troubles during the operation, and suppression of gastric peristalsis is one of them. In this study, we found that acupuncture stimulation on the abdomen, by its gastric relaxation effect, suppressed the gastric peristalsis and made the procedure easier than with the antispasmodic agent. 

The antispasmodic agent used in this study was glucagon, of which the gastrointestinal motility-suppressing effect was first reported by Stunkard et al. in 1972. AS Chernish et al. confirmed its usefulness as a pretreatment for gastrointestinal examination [17,18], it has been widely used as a pretreatment agent for the digestive tract. However, a number of adverse events such as shock (including hypotension), skin rash, arrhythmias, hypoxia, and increased blood pressure with the use of antispasmodics have been reported [6].

In this study, more frequent adverse events were observed in the MG than in the AG, and most of them were blood pressure fluctuations. The increase in the blood pressure is considered as a result of elevated activity in the sympathetic nervous system due to hypoglycemia as a reaction to the hyperglycemic effect of glucagon. This is considered as a result of overreaction of the parasympathetic nerve activity to the elevated sympathetic activity. In the AG, few patients had blood pressure fluctuations and it is considered that the burden on the endoscopist due to blood pressure fluctuation was lowered. 

### 4.1. Antispasmodic Effect of Acupuncture Stimulation

The migrating motor complex (MMC), which is a cyclical phenomenon of gastric motility of human in fasted state, consists of four phases: Phase I is a quiescent period with no contractions; phase II has intermittent, irregular low-amplitude contractions; phase III has short burst of regular high-amplitude contractions; and phase IV is a short transition period back to phase I. Contractions occur every 90–120 min periodically [19,20]. In our study, as our patients had been in the fasted state from the morning of the procedure day, we can assume that they had this four-phase MMC. There were 14 patients who showed no gastric peristalsis during ESD, and therefore they were not included in the study. It can be speculated that these 14 cases were in Phase I at the procedure, as the MMC occurred every 90 to 120 min. 

Acupuncture stimulates the skin and muscle sensory nerves and communicates through branch projections to the medulla oblongata, the cerebral aqueduct, and the hypothalamus via the spinal thalamus. Based on such nerve pathway it exerts a regulating action, an analgesic action, and an anti-stress action of the autonomic nerve [21].

The following mechanisms are considered to work when gastric motility is suppressed by acupuncture stimulation to the abdomen: The peristalsis inhibitory effect of acupuncture is a somatic visceral reflex caused by mechanically noxious stimuli to the skin and muscles of the abdomen which through afferent pathways are transmitted to the spinal cord segmentally [9].As the reaction is observed when the vagus nerve is cut, but then disappears when the splanchnic nerves including the sympathetic nerve that controls the stomach are cut, it is considered that the elevated sympathetic nerve activity is closely related to this effect. Additionally, it has been reported that acupuncture stimulation of muscles suppresses stomach motility more strongly than the stimulation of skin of the abdomen [9]. Furthermore, it has been clarified that intensity of the C fiber excitement is related [22]. It has been also experimentally found that acupuncture stimulation to the abdomen works via spinal cord and via the rostral ventrolateral medulla (RVLM) of the medulla oblongata, which is a region that performs sympathetic reflexes, acupuncture stimulation of the abdomen activates sympathetic nervous system via RVML, and gastric motility is suppressed by the action of catecholamine [23]. Thusly, gastric motility suppression by acupuncture stimulation to the abdomen is caused by the action through two regions: the spinal cord and medulla oblongata. 

In the acupuncture stimulation to CV12 that we performed in this study, as the depth of the acupuncture reached the white line of the abdominal muscle, and the fascia and white line were strongly stimulated by the acupuncture, it was considered that gastric motility was suppressed by the sympathetic nervous system via reflex and RVML.

### 4.2. Study Limitation

We also would like to mention that this study has some limitations. Firstly, this study was a quasi-randomized controlled trial. This pilot study was conducted as an unprecedented attempt to develop a complementary medicine for antispasmodics in ESD. As it was not clear how much antispasmodic effects in the upper gastrointestinal tract to be caused by acupuncture, we planned this quasi-randomization by assigning the first 10 cases to the acupuncture group with acupuncture stimulation and confirmed its antispasmodic effect. Therefore, confounding factors may not be adjusted because it is not a normal RCT, and the results should be considered conservatively.

Secondly, although subjects and the statisticians were not aware of the assignment, the endoscopists and the acupuncture practitioners were not masked. In particular, as the endoscopists knew which group each case was in, the possibility of introducing information bias cannot be denied. However, as the relaxation effect of the stomach caused by acupuncture was obvious, we consider that the sufficiently correct evaluation was performed. If acupuncture could serve as an alternative to upper gastrointestinal antispasmodics, non-inferiority studies should be done in the future.

## 5. Conclusions

We devised a usage of acupuncture to the abdomen as an alternative to antispasmodic drugs used during ESD of early gastric cancer. Acupuncture stimulation to the abdomen produced a relaxing action on the stomach and an antispasmodic effect was confirmed. In addition, compared to the drug, acupuncture stimulation to the abdomen was found to be significantly better in ease of surgery score and to have less fluctuation of blood pressure during the operation. It was considered that acupuncture to the abdomen could be an alternative to drugs. To extend our research, we may consider the idea that acupuncture stimulation could be used as an antispasmodic agent for duodenum or colon during endoscopic retrograde cholangiopancreationography (ERCP) or colon ESD.

## Figures and Tables

**Table 1:** Modified NIWA classification.

Evaluation	Class	State of Peristalsis
Very easy to operation	I	No peristaltic movement found and no problem with endoscopic operation. Equivalent to degree I (no peristaltic movement).
Easy to operation	II	Slight peristaltic movement found but no problem with endoscopic operation. Equivalent to degree II (slight peristaltic movement).
operation is slightly difficult	III	Some, although light, peristaltic movement found and some problem with endoscopic operation. Equivalent to degree III (mild peristaltic movement).
operation is difficult	IV	Peristaltic movement found and hard to perform endoscopic operation. Equivalent to degree IV (strong peristaltic movement).

**Table 2:** Baseline subject characteristics.

	MG (*n* = 30)	AG (*n* = 30)	*p* Value
Gender (M/F)	20/10	23/7	0.39
Age yr (IQR)	77.50 (72.3 to 82.0)	76.00 (67.0 to 81.5)	0.45
Height (m) (IQR)	154.40 (150.2 to 170.0)	158.80 (148.6 to 166.2)	0.31
Body Weight (kg) (IQR)	58.70 (48.9 to 66.5)	59.95 (52.3 to 65.5)	0.61
Body Mass Index (Kg/m^2^) (IQR)	23.90 (21.5 to 25.9)	23.70 (21.5 to 25.7)	0.63
Brinkman index (IQR)	161.00 (0.0 to 480.3)	0.00 (0.0 to 560.0)	0.67
ASA-PS *n* (%)			
Class I	18 (60.0)	23 (76.7)	0.46
Class II	7 (23.3)	4 (13.3)	
Class III	4 (13.3)	3 (10.0)	
Class IV	1 (3.3)	0 (0)	
Class V	0 (0)	0 (0)	
Complications *n* (%)			
Hypertension	17.00 (56.7)	18.00 (60.0)	0.79
Hyperlipidemia	3.00 (10.0)	6.00 (20.0)	0.28
Diabetes mellitus	5.00 (16.7)	9.00 (30.0)	0.22
Location *n* (%)			
Cardia	1 (3.3)	1.00 (3.3)	0.99
Body	7 (23.3)	6 (20.0)	
Incisura	6 (20.0)	6 (20.0)	
Anterum	16 (53.3)	17 (56.7)	
Macroscopic type, *n* (%)			
Type 0-I	3 (10.0)	2 (6.7)	0.92
Type 0-IIa	8 (26.7)	6 (20.0)	
Type 0-IIb	1 (3.3)	1 (3.3)	
Type 0-IIc	9 (30.0)	14 (46.7)	
Type 0-IIa + IIc	5 (16.7)	4 (13.3)	
Type 0-IIc + IIa	2 (6.7)	2 (6.7)	
Type 0-IIa + IIb	2 (6.67)	1 (3.3)	

ASA-PS: American Society of Anesthesiologists—physical status, MG: medication group, AG: acupuncture group. Data as medians and interquartile ranges (IQR), count data as numbers and percent.

**Table 3:** Changes in the modified NIWA classification and visual analogue scale.

	Baseline	After	Change from Baseline to Post Treatment Measurements	*p* Value
MNC (unit)				
AG (n 23) (IQR)	4.00 (3.0 to 4.0)	1.00 (1.0 to 1.0)	−2.00 (−3.0 to −2.0)	<0.0001
MG (n 23) (IQR)	4.00 (3.0 to 4.0)	2.00 (2.0 to 3.0)	−1.00 (−2.0 to −1.0)	
VAS (mm)				
AG (n 23) (IQR)	96.00 (77.5 to 100)	15.00 (7.0 to 23.0)	−73.00 (−87.0 to −59.0)	<0.0001
MG (n 23) (IQR)	80.00 (72.5 to 89.5)	29.00 (16.5 to 52.0)	−41.00 (−65.0 to −29.0)	

MNC: modified NIWA classification, VAS: visual analogue scale, MG: medication group, AG: acupuncture group. Data as medians and interquartile ranges (IQR).

**Table 4:** Changes in the other outcome and procedure complications.

	MG (*n* = 23)	AG (*n* = 23)	*p* Value
Procedure time (minute) (IQR)	53.00 (44.0 to 76.0)	93.00 (70.5 to 138.0)	0.005
Anesthesia time (minute) (IQR)	51.00 (40.0 to 72.0)	85.00 (65.5 to 132.0)	0.003
Resected specimen area (mm^2^) (IQR)	480.00 (358.5 to 621.0)	598.00 (428.0 to 820.5)	0.09
Resection en bloc (%)	23 (100)	23 (100)	N.A
Sedative medication			
Midazolam (mg) (IQR)	16.00 (14.0 to 20.0)	18.00 (14.0 to 20.0)	0.25
Propofol (mg) (IQR)	0.00 (0.0 to 6.5)	0.00 (0.0 to 9.0)	0.61
Pentazocine (mg) (IQR)	1.00 (0.5 to 1.0)	1.00 (0.5 to 1.0)	0.89
Procedure Complications *n* (%)			
Bleeding			
Inoperative	2.00 (8.70)	1.00 (4.35)	0.55
Postoperative	0 (0)	0 (0)	N.A
Perforation			
Intraoperative	1.00 (4.35)	0 (0)	0.31
delay	0 (0)	0 (0)	1.00
Blood pressure fluctuation	14.00 (60.86)	2.00 (8.70)	0.001
Emergency surgery	0 (0)	0 (0)	N.A
Aspiration pneumonia	0 (0)	0 (0)	N.A

MG: medication group, AG: acupuncture group, N.A: not applicable. Data as medians and interquartile ranges (IQR), count data as numbers and percentages.

## Data Availability

The complete datasets generated during the current study are available from the corresponding author on reasonable request.

## Supplementary Materials

The following are available online at https://www.mdpi.com/article/10.3390/jcm10143050/s1. The study protocol (File S1) and data set of primary and secondary outcome measures (File S2) used in this study are presented.
