# Supplementary material for: Pilot Study of Acupuncture’s Antispasmodic Effect on Upper Gastrointestinal Tract during Endoscopic Submucosal Dissection for Early Gastric Cancer: Controlled Clinical Trial
Source: J Clin Med. 2021 Jul 9;10(14):3050. doi: 10.3390/jcm10143050 (PMC8305036; doi:10.3390/jcm10143050)
Supplement: Supplementary file 1 [file jcm-10-03050-s001.zip › Data set of primary and secondary outcome measures (S2).pdf]

Data set of primary and secondary outcome measures.

Group	Baseline MNC	Post MNC	diff MNC	Baseline VAS	Post VAS	diff VAS
MG	4	3	-1	54	54	0
MG	4	4	0	95	95	0
MG	3	2	-1	63	22	-41
MG	3	1	-2	78	3	-75
MG	4	1	-3	100	0	-100
MG	4	3	-1	88	88	0
MG	3	1	-2	70	5	-65
MG	4	2	-2	75	16	-59
MG	3	2	-1	80	43	-37
MG	4	2	-2	90	28	-62
MG	3	2	-1	70	56	-14
MG	3	1	-2	95	5	-90
MG	4	2	-2	100	17	-83
MG	3	1	-2	89	16	-73
MG	3	2	-1	77	19	-58
MG	3	2	-1	63	30	-33
MG	3	2	-1	70	31	-39
MG	3	2	-1	80	29	-51
MG	4	3	-1	75	60	-15
MG	4	3	-1	83	48	-35
MG	4	3	-1	79	50	-29
MG	4	2	-2	83	26	-57
MG	4	3	-1	97	63	-34
AG	4	2	-2	98	30	-68
AG	3	1	-2	80	23	-57
AG	4	1	-3	100	23	-77
AG	4	2	-2	95	34	-61
AG	4	1	-3	100	20	-80
AG	4	1	-3	100	0	-100
AG	3	1	-2	74	21	-53
AG	3	1	-2	75	11	-64
AG	4	1	-3	100	0	-100
AG	4	1	-3	96	9	-87
AG	4	1	-3	100	15	-85
AG	4	1	-3	100	0	-100
AG	3	2	-1	84	40	-44
AG	3	1	-2	69	10	-59
AG	3	1	-2	72	10	-62
AG	4	1	-3	97	16	-81
AG	3	2	-1	75	18	-57
AG	4	1	-3	91	0	-91
AG	3	1	-2	90	5	-85
AG	4	1	-3	100	0	-100
AG	3	1	-2	75	13	-62
AG	4	2	-2	100	42	-58
AG	4	1	-3	100	27	-73

MG:Medication group, AG: Acupuncture group,
MNC: modified NIWA classification, VAS: visual analogue scale
