# Supplementary material for: Pilot Study of Acupuncture’s Antispasmodic Effect on Upper Gastrointestinal Tract during Endoscopic Submucosal Dissection for Early Gastric Cancer: Controlled Clinical Trial
Source: J Clin Med. 2021 Jul 9;10(14):3050. doi: 10.3390/jcm10143050 (PMC8305036; doi:10.3390/jcm10143050)
Supplement: Supplementary file 1 [file jcm-10-03050-s001.zip › The study protocol (S1).pdf]

Study protocol (Original in Japanese)

1. Title

Pilot Study of Acupuncture's Antispasmodic Effect on Upper Gastrointestinal Tract during Endoscopic Submucosal Dissection for Early Gastric Cancer: Controlled Clinical Trial

2. Researchers

(1) Principal investigator

Prof. Goro Shibukawa

(2) Main researcher

Assoc. Prof. Masao Suzuki

(3) Associate researchers

Prof. Atsushi Irisawa

Prof. Tadamichi Mitsuma

Research Assoc. Tsuneteru Hoshi

Research Assoc. Akane Yamabe

Research Assoc. Mariko Fujisawa

Research Assoc. Akira Funakubo

Hospital Assoc. Ryo Igarashi

Graduate Student Researcher Takumi Maki

Research Assoc. Taiga Furuta

Research Assoc. Takumi Kayo

Prof. Naoto Ishizaki

3.Background

For early gastric cancer, endoscopic submucosal dissection (ESD) is currently the treatment of choice and to prevent adverse events such as bleeding and perforation during ESD, the inhibition of gastrointestinal tract peristalsis (spasmolysis here after) is conducted. Medicines such as "anticholinergic drug" or "glucagon" are used for spasmolysis. However, these medicines may be contraindication or should be used carefully when the patient has underlying disease (such as glaucoma, hypertension, prostatomegaly, heart disease, diabetes, etc.). Mint preparation has been used for spasmolysis recently, but some endoscopists do not use mint preparation since not only essential oil constituent hampers the effect of indigo liniment of gastric mucosa and but also due to its limited duration of the spasmolysis effects.

It is well known that these underlying diseases are increasing in these years. Patients with those underlying diseases would have to take a great risk when such spasmolysis medications are used on them and physicians would also face great stress of possible accident during operation. Nationwide research on adverse events related to utilization of gastrointestinal endoscopy (Gastroenterol Endosc 2010 (52) 95-103) revealed that there were many cases of procedural accidents due to spasmolysis medications and brought up problems such as interruption and stoppage of ESD due to sudden change of condition. Nevertheless, there is no other procedure other than such medications. Therefore, it is needed to develop and establish a procedure not using

such spasmolysis medications while the number of patients with such underlying diseases should increase.

On the other hand, the“Zhongwan point”used in acupuncture treatment is said to be effective for the convulsions of the stomach. It has been reported recently （Auton Neurosci 2008 (138) 91-8）that the effects of acupuncture stimuli to the Zhongwan point to inhibit the movement of stomach was experimentally verified. Moreover, the spasmolysis effects by acupuncture stimuli, which utilize the reaction of the autonomic nervous system, are considered safe and have no side effects like medications. Since there has been no report of spasmolysis effects by acupuncture stimuli in case of ESD, this study would be the very first one in the world.

[Purpose]

Therefore, we will try to establish the procedure of spasmolysis using acupuncture stimuli to abdomen as a safe and secure way of spasmolyis by taking advantage of the mechanism which are innate to human body and not relying on medications and also will try to consider feasibility as well as safety of applying acupuncture stimuli to the Zhongwan point during ESD.

4. Principle of research target selection

[Inclusion criteria at the start of the clinical trial]

Inclusion criteria are: (A) age > 40 years and diagnosed as early-stage gastric cancer, irrespective of H. pylori infection. Early-stage gastric cancer was pathologically diagnosed using endoscopy prior to ESD. The criteria of early-stage gastric cancer are: (1) An intramucosal intestinal-type cancer without ulcerative lesion, regardless of tumor size; (2) intramucosal intestinal-type cancer with ulcerative lesion, ≤ 3 cm in size; and (3) intramucosal diffuse type cancer ≤ 2 cm in size without ulcerative lesion.

(B) Those who are in stable condition without any symptoms of infectious diseases such as fever or respiratory symptoms, showed no rapid deterioration of cancer-related symptoms and with no changes in the medication at least a week prior to the date of the ESD.

(C) Patients who has sufficient cognitive ability to understand the study protocol.

[exclusion criteria at the start of the clinical trial]

Exclusion criteria are patients with (1) history of partial gastrectomy; (2) severe uncontrollable diabetes or severe cardiovascular disease; (3) advanced stage gastric cancer; and (4) lack of informed consent.

[Sample size]

1. Number of cases to be aimed at is 60.
2. Sample size is obtained based on the prier research (JJSAM. 2004; 54(4): 779-784.) by using the test of difference (two-sided) in the mean values of effects in inhibition of gastrointestinal tract peristalsis between acupuncture stimulated group and medication group during ESD. The difference between the groups in inhibition of gastrointestinal tract peristalsis (Modified NIWA classification: MNC) is indicated asτ, standard deviation

asσ.　When the significance level isα and power is 1-β, sample size of one group N is shown as

$$N = \frac{2\sigma^2\left(z_{1-\beta} + z_{1-\alpha/2}\right)^2}{\tau^2}$$

Here, z_ε the percentile of the standard normal distribution.
Previous studies (JJSAM. 2004; 54(4): 779-784.) have reported that gastric peristalsis was suppressed by about 1.8 points in MNC when acupuncture was applied to the abdomen (CV12) during upper gastrointestinal endoscopy procedure. Furthermore, the difference of MNC change between the acupuncture group and the medication group was 0.2 points. Since the previous study was a non-RCT and it found no significant difference between medication group and acupuncture group, we took a fairly conservative estimation of MNC difference as 1 and the standard deviation as 1.
We show in the Table 1 the calculated sample size for each standard deviation 0.5, 0.75, 1.0, and 1.25 with various powers from 50% to 90% at the significance level 5%.
Thus, the required sample size was calculated to be 46 patients (MG 23 and AG 23) to detect a minimal difference of MNC means = 1 with SD=1 at a significance level of 0.05(α=0.05) with a power of 0.9(β=0.1).
This study requires recruitment of 30 patients per group in anticipation of a certain dropout.

τ	Power	Standard deviation			
		0.5	0.75	1.0	1.25
1	90	13	26	**46**	68
	80	11	20	34	52
	70	9	17	27	41
	60	8	14	22	33
	50	7	11	18	27

[Method of patient recruitment]
Relevant patients are recruited by the physician in charge.
[Period of study]
December 2015 – December 2016 (12 months)
[Research facilities]
1. Fukushima Medical University, Aizu Medical Center
Address: 21-2 Maeda, Tanisawa, Kawahigashi, Aizuwakamatsu City Fukushima, 969-3492 Japan
2. Meiji University of Integrative Medicine
Address: 6-1 Hinotani, Honoda, Hiyoshicho, Nantan City, Kyoto, 629-0301 Japan

5.Research Method
（１）Procedures, Chart of interrelationship

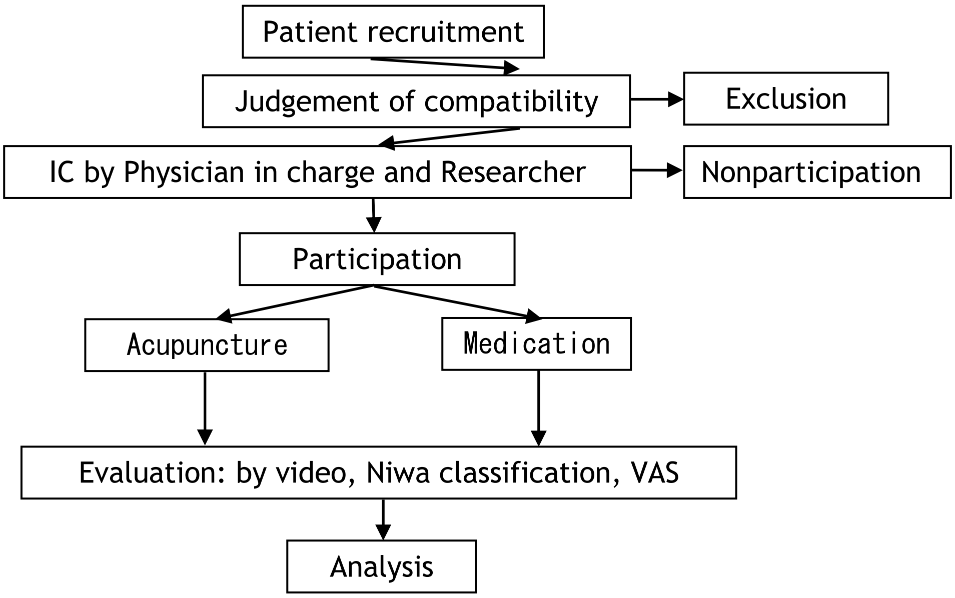

(2) Explanation on research method
Examination design: Quasi-randomized parallel-group comparison test
Acupuncture group (AG): Group whose spasmolysis is inhibited using acupuncture
Medication group (MG) Group whose spasmolysis is inhibited using usual medication
* Acupuncture is administered at Zhongwan point (CV12) as shown in the figure. Acupuncture needles used are disposable needle of 40mm-60mm in length and 0.25mm in thickness. Stimulation procedure: After inserting a needle at Zhongwan point (in the middle between naval and xiphoid process on the median line), push forward up to white line area of rectus abdominis muscle white line of the, then turn the needle to give stimulation.

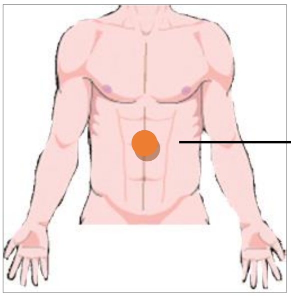

Figure：Zhongwan point (CV12)

*Medication used:
Glucagon are used (Glucagon G(1mg/ml) intravenous administration).
* Initial evaluation of the peristaltic movement is observed at the antrum including the pyloric ring. In case gastrointestinal tract peristalsis is found, medication or acupuncture stimulation is given when the Niwa classification is no less than 2.
* Duration of intervention: during ESD procedure
* Evaluation item
A) Primary outcome measure: evaluate the ease of ESD by endoscopist using revised Modified Niwa classification (table1)
B) Secondary outcome measure
Evaluation of the ease of ESD by endoscopist using Visual analogue scale (VAS).

VAS is a 100mm horizontal scale of which the left end (0mm) represents "the easiest" and the right end (100mm) "the most difficult".

*The Primary outcome measures and Secondary outcome measures were obtained by independent evaluators.

C) Other outcome measure: Indices of adverse events

Other outcome measures related to complications are; (1) bleeding (intraoperative and postoperative), perforation (intraoperative and delay) due to ESD. Here, bleeding during the procedure is defined as when bleeding continued even after coagulation with normal hemostat, and clipping or alcohol local injection had to be performed. Bleeding after treatment is defined as when hemostasis at the excised site had to be performed by upper gastrointestinal endoscopy because of hematemesis, melena, or shock.

Perforation is defined as when perforation was clearly confirmed during the operation and endoscopic clip closure is performed. Delayed perforation is defined as when peritonitis is suspected and free air was found by abdominal X-ray or CT scan.

Also, blood pressure fluctuation is monitored during procedure.

Blood pressure fluctuation is defined as greater than 30% increase or decrease from the baseline value (base line value is the blood pressure during a 10-minute rest before the procedure) lasting for 3 minutes or more within 5 minutes after the intervention with antispasmodics or acupuncture.

The presence or absence of aspiration pneumonia after treatment is evaluated comprehensively by not only respiratory symptoms (cough, sputum, dyspnea, chest pain), and fever but also findings by images of chest X-ray or chest CT, and sputum examination and blood culture.

In accordance with the ASA Physical Status Classification System (ASA-PS), the operation time, anesthesia time, total amount of sedative medication, and resection diameter were also evaluated.

Table 1　Modified Niwa Classification

Evaluation	Degree	Peristaltic movement
Very easy to operation	1	No peristaltic movement found and no problem with endoscopic operation.<br>Equivalent to degree I (no peristaltic movement).
Easy to operation	2	Slight peristaltic movement found but no problem with endoscopic operation. Equivalent to degree II (slight peristaltic movement).
Somewhat difficult to operation	3	Some, although light, peristaltic movement found and some problem with endoscopic test. Equivalent to degree III (mild peristaltic movement).
Difficult to operation	4	Peristaltic movement found and hard to perform endoscopic operation. Equivalent to degree IV (strong peristaltic movement).

- Criteria of Intervention Cancellation

Cases to be considered as intervention cancellation
1. when a patient’s condition changes suddenly during operation (sudden drop of blood pressure, lowered oxygen saturation, significant change of heart rate)
2. when a patient’s condition changes due to acupuncture stimulation (sudden drop of blood pressure, lowered oxygen saturation, significant change of heart rate)
3. when the physician in charge of ESD operation considers further acupuncture stimulation to a patient is not appropriate

- Procedure of Study Stoppage (cancellation)
  1. During the study period, in case the physician in charge or the physician operating ESD recognizes that the safety of a patient cannot be secured due to the acupuncture stimulation, the principal investigator and the main researcher shall be consulted and can decide to cancel the study.
  2. If the cancellation of the study is decided, all who are involved in this study should be notified within one week and the study is cancelled.

- Protocol of the Study

Quasi-Randomization: The first 10 patients were assigned to the acupuncture group (AG), the next 10 patients to the medication group (MG), then the next 10 patients into AG, and so on. This alternate assignment procedure was repeat ed until 60 cases (30 cases in each group) were enrolled.

1. The physician in charge evaluates whether the said patient (with possible gastric cancer in its early stage) fulfills the selection criteria.
2. The physician in charge confirms the patient’s intention orally whether he/she is “willing” or “not willing” to participate in this study. Subsequently, the physician in charge or the clinical researcher gives explanation of the study along with written documents to the patient who is willing to participate to reconfirm it. When the patient’s willingness is confirmed, informed consent is obtained by a letter of consent.
3. During the EDS of the said patient, if the peristaltic movement of the stomach is found, acupuncture stimulation or medication will be administered.
(In case gastrointestinal tract peristalsis is found, medication or acupuncture stimulation is given when the Niwa classification is no less than 2.)
On the other hand, if the peristaltic movement of the stomach is NOT found, ESD is performed without using acupuncture stimulation or medication. In this case, it would be explained to the patient that neither acupuncture stimulation or medication were administered after ESD as an exception.
4. When the ESD is done, “peristalsis inhibitory effect” and “ease of EDS” are evaluated using the Modified Niwa Classification and VAS by operating physician.
5. All the evaluation date are analyzed by two biostatisticians:
Naoto Ishizaki, Course of Acupuncture and Moxibustion, Faculty of Health Sciences, Tsukuba University of Technology,
Takumi Kayo, Fukushima Medical University, Aizu Medical Center

[Statistical Analysis]

The following analyses are to be performed according to Full Analyze set.

In this study, we set two analysis groups, FAS (Full Analyze set) and PPS (Per protocol set), and analyze the data for each analysis group.

The main analysis group in this study is FAS. The definitions of FAS and PPS in this research are defined as follows.

FAS: All patient groups who were assigned, excluding dropped out patients.

PPS: A group of FAS that excludes patients who did not receive acupuncture or medication.

Statistical analysis of this study was conducted by an independent two biostatisticians (K.T., I.N.).

For the baseline evaluation items, distributions of the measurements at the baseline are shown.

For main evaluation item and secondary evaluation items, means and standard deviations of the difference between baseline and endpoint are calculated. Proportions are calculated for count variables.

Primary outcome measure and secondary outcome measure, time lengths of surgery and anesthesia, amount of sedative medication used, and excision diameter, difference between the groups are evaluated, with significance level of 0.05, using Mann-Whitney test or Chi-square test for count variables.

An independent data entry person prepares the whole dataset and encrypts the patient ID so that the individual cannot be identified. In order to avoid bias, the biostatisticians perform the statistical analysis with grouping information masked.

6. Expected research results and academic contributions

Endoscopic surgery (ESD, etc.) as an alternative to surgical operation is now becoming the mainstream choice of treatment for early gastric cancer and this trend is expected to accelerate. At the same time, various drugs are often used in such endoscopic surgeries, and antispasmodics are used as a pretreatment in ESD. Unfortunately, a fairly large number of adverse events due to side effects of these antispasmodics have been observed, therefore, contraindication or careful administration is required especially when the patient has a basic disease (such as heart disease and diabetes). However, without any alternative methods to replace these drugs, and if ESD is not possible, the patient has to go through surgical operation with greater physical, psychological, and financial burden on the patient, and also on society with greatly increased medical costs. Expected results of our research are to indicate that acupuncture stimulation during ESD is effective in suppressing gastric peristalsis as much as or better than existing drugs, makes ESD safe without side effects, and can minimize the physical, psychological, and financial burden on the patient. In addition, our method could be used not only the upper gastrointestinal tract but also the lower gastrointestinal tract, and antispasmodic effect of acupuncture can be applied to early colorectal cancer operation. Therefore, we believe that the results of this study have a high academic significance as an adjunct therapy for surgery and highly contribute to the society.

7.Basis for scientific rationality of research

Previous studies have reported that acupuncture stimulation to the abdomen causes gastric relaxation. Also, it has been demonstrated pharmacologically that catecholamine ß receptors and nicotine receptors are involved since the administration of guanesidine, propanol, and hexamethonium eliminates acupuncture-induced gastric relaxation. Its mechanism is considered as this: acupuncture to the abdomen stimulates the skin and muscles at the stimulation site, and this stimulation is input from the dorsal horn of the spinal cord and reaches the medullary Rostral Ventral Lateral Medulla (RVLM), which informs presynaptic sympathetic neurons, leads to the release of catecholamines, which makes the stomach relax. (Dig Dis Sci. 2003;48:59-68.) Thusly, the acupuncture stimulation to the abdomen conducted in this study is pharmacologically proved and the mechanism is elucidated. Therefore, it is considered that the scientific rationality is well secured.

8. Ethical considerations in research

1. Informed consent
   Research contents are explained to each participant using written material and a written informed consent to participate is given by the participant.
2. Protecting the human rights of research subjects
   - This study is intended only for those who have received an explanation of this study and agreed to participate.
   - If the subject who has given consent requests to stop participating after the start of this study, the subject's will shall be respected.
   - Participation/non-participation in this study is based on the free will of the subject, and no disadvantage is affected for those not agree or cancel the participation.
3. How to seek understanding from research subjects and obtain consent
   a. The explanation of the contents of this study will be given to the patient by the physician in charge or the main researcher during the ESD explanation.
   b. Research contents will be explained to the patient with written material (Research protocol).
   c. As for the method of obtaining consent, the consent form for this study is used, and the consent is obtained with the patient's signature.
4. Handling of personal information

   The samples and data used for this research will be anonymized so that they can maintain the reliability of research analysis.

   Specifically, data is created by assigning a number to the medical information for each individual, and a correspondence table of data and numbers anonymized by deleting the personal information is created and recorded in an external storage medium.

   The anonymized data will be stored by the researcher, and the correspondence table will be stored by the main researcher (personal information protection manager of this research) in the locked storage at each research site.

The computer that stores the anonymized data and the correspondence table is separated from other computers.

5. Dangers or disadvantages to the subject that this study may result

   Pain and internal bleeding due to acupuncture may be the disadvantages to the subjects that may result from this study. Since ESD is performed using sedatives, acupuncture stimulation usually does not cause pain. However, although the pain of the acupuncture part (abdomen) may be felt after the effect of the sedative is disappeared, it usually disappears in about one day, and no medical treatment is required. In addition, a slight internal bleeding may occur in the acupuncture site, but usually it does not require medical treatment because it usually resolves spontaneously within 1 to 2 weeks.

6. Response when the matters described in (5) above actually occur or can be predicted to occur

   If an adverse event occurs during abdominal acupuncture and medical treatment is required, appropriate treatment (prescription of analgesic or poultice) should be taken. If internal bleeding is predicted, compression hemostasis is performed after ESD.

7. Correspondence to contacts from research subjects and related persons

   As a general rule, the main researcher (Masao Suzuki) handles any contacts from research subjects and related parties, but in the case of specialized consultations on gastric cancer and endoscopy, it will be handled by the Gastroenterologist in the joint research team.

8. Correspondence about medical care provision

   If an unexpected serious adverse event occurs in this study, appropriate medical care will be provided based on the judgment of the physician in charge and the main researcher.

9. How to raise research funds

1. Research funds

   This research was funded by the Grants-in-Aid for scientific research from the Japan Agency for Medical Research and Development (AMED).

2. Conflicts of interest

   None.
